# Dermatomyositis Flare With Immune Checkpoint Inhibitor Therapy for Melanoma

**DOI:** 10.7759/cureus.14387

**Published:** 2021-04-09

**Authors:** Rachel Thomas, Hamish Patel, Joshua Scott

**Affiliations:** 1 Internal Medicine, Wright State University Boonshoft School of Medicine, Dayton, USA; 2 Rheumatology, Brooke Army Medical Center, Fort Sam Houston, USA; 3 Rheumatology, Wright-Patterson Medical Center, Dayton, USA

**Keywords:** metastatic melanoma, myositis, immune checkpoint inhibitors (icis), immune-related adverse effects, nivolumab, myositis flare, dermatomyositis

## Abstract

Immune checkpoint inhibitor (ICI) medications have seen expanded use in the management of numerous malignancies. These therapies encompass a unique spectrum of immune-related adverse events (irAEs). Rheumatological irAEs from ICIs have been described in various case reports; however, limited literature exists regarding the treatment of patients with pre-existing myositis. We describe a case of myositis flare after initiation of nivolumab for metastatic melanoma in a patient who had previously achieved remission of dermatomyositis.

## Introduction

Immune checkpoint inhibitors (ICIs) have seen expanded use in the management of numerous malignancies. These medications were first approved for use in metastatic melanoma and are now approved for use in other malignancies including but not limited to non-small cell lung cancers, urothelial cancers, gastric adenocarcinoma, and Hodgkin’s lymphoma [1]. While these therapies have provided significant survival benefit, they encompass a unique spectrum of immune-related adverse events (irAEs). The most common irAEs are dermatologic and gastrointestinal in nature; however, endocrine, hepatic, pulmonary, and neurologic side effects have also been reported [1-4]. Rheumatological irAEs from ICIs have been described in various case reports. However, limited literature exists on the use of ICIs in patients with pre-existing dermatomyositis, and completion of ICI therapy after a myositis flare is uncommon. Here we describe a case of flare of dermatomyositis after initiation of nivolumab for metastatic melanoma in a patient who had previously achieved myositis remission on rituximab.

## Case presentation

**Figure 1:**
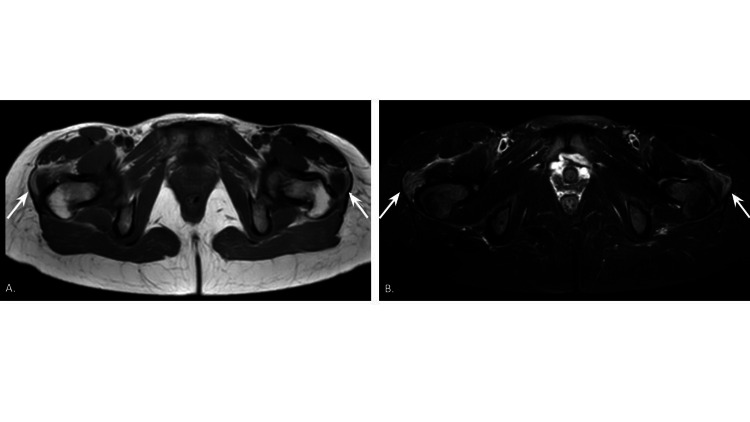
MRI axial T1 (A) and T2 (B) images of the bilateral lower extremities during dermatomyositis flare on nivolumab therapy. Muscle edema in the bilateral gluteus minimus and gluteus medius muscles was noted.

A 36-year-old female presented to the rheumatology clinic with progressive weakness and rash during her second pregnancy. The patient was diagnosed with dermatomyositis based on decreased proximal muscle strength (3/5) in the bilateral lower extremities, Holster sign rash, Gottron papules, elevated creatine kinase (CK) (564 U/L), elevated aldolase (11.8 U/L), elevated inflammatory markers, high antinuclear antibody titer (>1:1280), and positive transcriptional intermediary factor 1-gamma (TIF1-γ). Cancer screening including mammography, breast ultrasound, transvaginal ultrasound, pelvic MRI, and skin examination was negative for evidence of malignancy. She was subsequently treated with 5-20 mg of prednisone daily during her pregnancy. However, post-partum, the patient had a recurrence of her myositis symptoms with corresponding changes on her MRI of the bilateral lower extremities. Treatment with prednisone and mycophenolate mofetil failed to control her disease. Rituximab was initiated for further steroid-sparing. Initial rituximab dosing was two 1-gram infusions 14 days apart. Rituximab was continued for the maintenance of remission dosed at 500 mg infusions separated by 14 days every six months. Four months after her second maintenance dose of rituximab, the patient was diagnosed with stage IIIA melanoma after biopsy of an evolving pigmented lesion on her left thigh. The patient underwent wide local excision and sentinel node dissection. Two out of three nodes were positive for micro-metastasis; however, positron emission tomography (PET) was negative for distant metastasis. The patient elected to undergo adjuvant treatment with nivolumab. After the second infusion, the patient experienced recurrent myositis symptoms including severe fatigue, proximal weakness, Gottron papules, and elevated CK (1,071 U/L). MRI of the proximal bilateral lower extremities again demonstrated inflammatory changes, consistent with her prior dermatomyositis episodes (Figure 1). The patient was treated per the American Society of Clinical Oncology guidelines with 20 mg of prednisone tapered over six months [1]. She was able to complete the full 12 months of nivolumab therapy without any further steroids or flares of her dermatomyositis.

## Discussion

ICIs block specific pathways in the immune checkpoint cycle, resulting in increased T-cell activation. Proteins typically expressed on T-cells known as cytotoxic T-lymphocyte-associated-4 (CTLA4) and programmed cell death protein 1 (PD-1) bind with their respective ligands, CD80/CD86 and programmed cell death ligand 1 (PD-L1), causing T-cell deactivation. Cancer cells mimic these ligands in order to evade and dampen the natural immune response. The use of ICIs, by blocking either CTLA4, PD-1, or PD-L1, results in an upregulation of the immune response, which helps target and destroy tumor cells [5]. However, due to non-specific T-cell stimulation, these therapies result in various immune-related adverse events ranging from mild to severe.

ICI use among individuals with underlying autoimmune conditions can be limited because of not only their side effect profile but also the ability to dampen the aforementioned T-cell regulation. While it is true that most irAEs manifest as dermatologic or gastrointestinal issues, emerging literature and case reports have noted a growing number of rheumatologic irAEs [6-7]. The most common of these symptoms include primary arthritis, sicca symptoms, and polymyalgia rheumatica-like syndromes [4]. A retrospective study by Abdel-Wahab et al. noted that 75% of patients with a preexisting autoimmune disease developed an irAE with ICI treatment [8]. More than half of these patients experienced a flare of their prior disorder, whereas less than one-third developed a different irAE. In a more recent study by the Dutch Melanoma Treatment Registry, the rate of irAE was 30% irrespective of the presence of an underlying autoimmune condition [9].

Myositis is a rare irAE of ICI therapy, with few cases reported. A review of prior case series showed the development of myositis in a few patients without an underlying autoimmune condition [10-11]. The greater concern lies with the severe or fatal complications of myositis. Progression of the inflammatory myopathy can result in end organ damage, with the most severe causing myocarditis. One particular literature review from Sweden revealed that 29 of 180 cases of myositis were complicated by myocarditis, leading to a significantly increased mortality rate (51.7% versus 14.9% in ICI-related myositis without myocarditis) [12].

## Conclusions

ICI therapy is associated with a unique spectrum of adverse events due to inhibition of the usual T-cell regulatory cycle. For those with an underlying autoimmune condition, the increased T-cell activation secondary to ICI commonly triggers a disease flare. While most rheumatological irAEs can be managed with corticosteroid therapy, the risks and benefits of initiating or continuing ICI therapy must be carefully weighed. ICI therapy is commonplace in oncology, and understanding the spectrum of irAEs is paramount in management. The case in this report not only highlights the ability to successfully complete ICI therapy in a patient with preexisting dermatomyositis but also illustrates the importance of recognizing and treating irAEs early as complications can be swift and severe.
